# Microbial community structure characteristics among different karst aquifer systems, and its potential role in modifying hydraulic properties of karst aquifers

**DOI:** 10.3389/fmicb.2022.1054295

**Published:** 2023-01-17

**Authors:** Zuobing Liang, Shaoheng Li, Zhuowei Wang, Rui Li, Zhigang Yang, Jianyao Chen, Lei Gao, Yuchuan Sun

**Affiliations:** ^1^School of Geography and Planning, Sun Yat-sen University, Guangzhou, China; ^2^Key Laboratory of Groundwater Sciences and Engineering, Ministry of Natural Resources, Shijiazhuang, China; ^3^China Institute of Water Resources and Hydropower Research, Beijing, China; ^4^Department of Hydraulic Engineering, Tsinghua University, Beijing, China; ^5^South China Botanical Garden, Chinese Academy of Sciences, Guangzhou, China; ^6^Chongqing Key Laboratory of Karst Environment, School of Geographical Sciences, Southwest University, Chongqing, China

**Keywords:** karst aquifer, subsurface microbiology, heat tracer, isotopes, hydraulic properties

## Abstract

Little is known about how microbial activity affects the hydraulic properties of karst aquifers. To explore the potential impacts of microbial activity on the hydraulic properties of karst aquifers, microbiological analysis, heat tracer, isotope (dissolved inorganic carbon isotope, δ^13^C_DIC_) and aqueous geochemical analyses were conducted at six monitoring wells in Northern Guangdong Province, China. Greater hydraulic conductivity corresponded to a low temperature gradient to an extent; the temperature gradient in karst groundwater aquifers can reflect the degree of dissolution. Higher HCO_3_^−^ concentrations coupled with lower d-excess and pH values at B2 and B6 reflect potential microbial activity (e.g., *Sulfuricurvum kujiense*) causing carbonate dissolution. Microbial activity or the input of anthropogenic acids, as evidenced by significantly more positive δ^13^C_DIC_ values, potentially affect carbonate dissolution in deep karst aquifers, which eventually alters hydraulic properties of karst aquifer. However, more direct evidence is needed to quantify the effects of microbial activity on carbonate dissolution in karst aquifers.

## 1. Introduction

Karst regions cover 7 ~ 12% of the Earth’s continental area, and their aquifers are a source of drinking water for almost one quarter of the global population (Ford and Williams, 2007). However, karst aquifers have complex characteristics that make them very different from other aquifers, because they are self-developing, dissolved bedrock constituents are transported through and out of the system (Ford and Williams, 2007), and this unique property of the aquifer results in difficulty developing and utilizing karst groundwater (Goldscheider and Drew, 2007).

Karst aquifers can be modified by external factors, such as climate change (Loáiciga, 2009), which alter the flux of meteoric water into the system and change the pressure or temperature imparted by vertically migrating fluids and internal processes (Corbella and Ibáñez, 2003), like chemical reactions (Loáiciga, 2009) and microbial activity (Engel et al., 2004a,b). In addition, mine water produced by mining activities also accelerate the dissolution rate of rocks in karst aquifers (Zhao et al., 2020; Liu et al., 2022), and seepage-damage effect in fractured rocks of karst aquifers may also produce great influences on the conductivity of karst aquifer (Zhao et al., 2021). More studies of microbial control of karst processes have focused on the sulfidic, saline water zones of karst aquifers (Engel and Randall, 2011; Gray and Engel, 2013). However, few reports have been focused on carbonate dissolution in freshwater aquifers. Lianjiang River Basin (LRB) is located in the north of Guangdong Province, China, characterized by high spatial hydrogeological heterogeneity. The LR system belongs to the trellis drainage, extending from the northwest to the southeast of the drainage basin. In the northwest of the LRB, karst aquifers have low permeability and low water richness, fissures are not developed; in the southeast of the drainage basin, karst aquifers have high permeability, joints and fissures are relatively developed, and dissolution is obvious.

In the present study, we determined the n ~ Alkanes biomarkers, groundwater temperature, dissolved inorganic carbon isotope (δ^13^C_DIC_), microbial community and hydrochemical characteristics of groundwater from the LRB to elaborate the following objectives: (1) ascertaining the structures of microbial communities in different karst aquifers; (2) exploring the effects of biogeochemical processes in modifying hydraulic properties.

## 2. Materials and methods

### 2.1. Well selection and geochemical analyses

**Figure 1:**
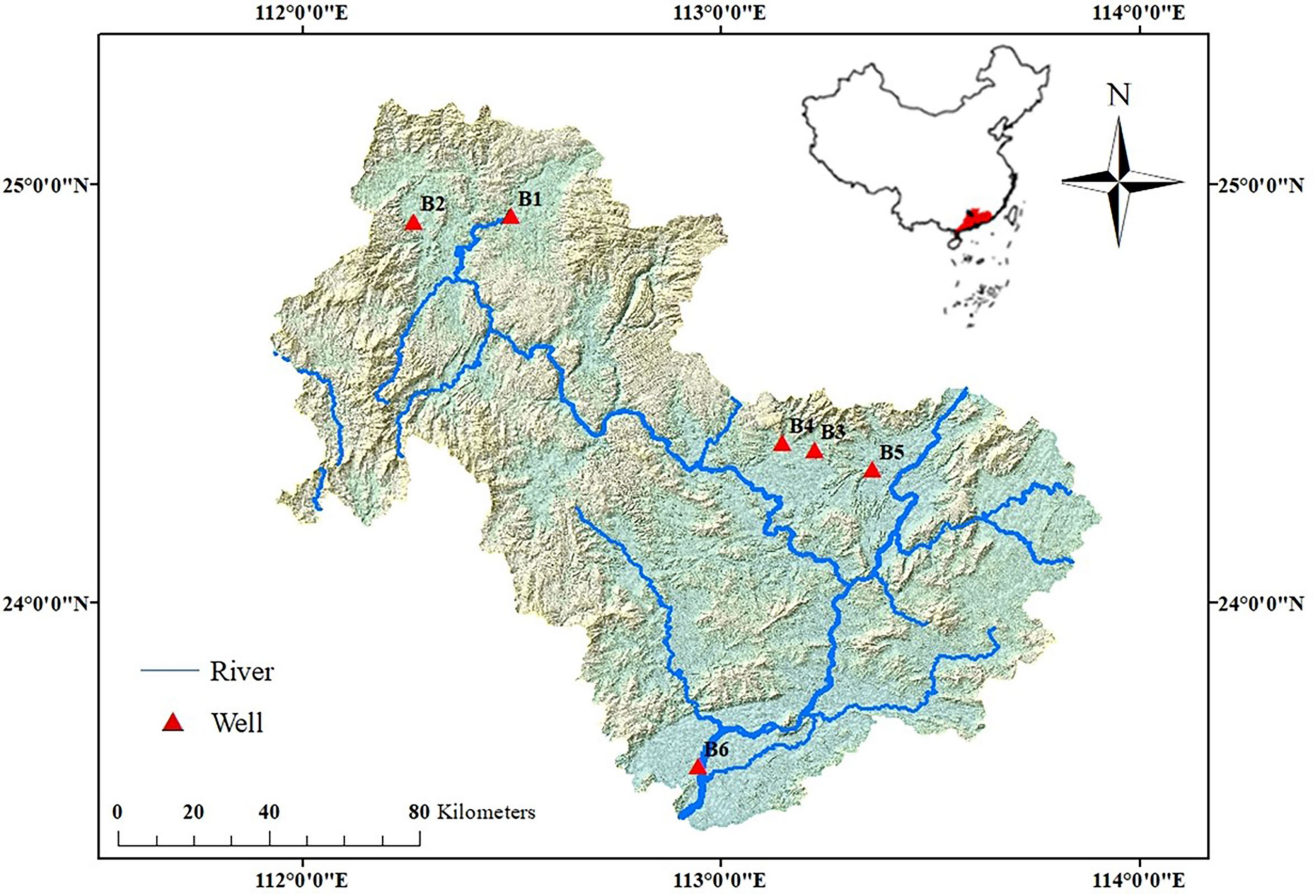
Map of the sampling sites.

Six monitoring wells are located in the Lianjiang River basin: B1 and B2 are upstream, B3–B5 are in midstream, and B6 is downstream (Figure 1). B1 is in an area of Cretaceous Nanxion calcareous mudstone; B2 is in an area of Devonian (Baqi/Liujiang) carbonate; B3 is in an area of Devonian Rongxian carbonate; B4 and B5 are in an area of Carboniferous Shidengzi carbonate; and B6 is in an area of Devonian Tianziling carbonate. More information about the lithology of selected boreholes was seen in attachment files (Supplementary Figure S1; Supplementary Table S1).

Groundwater from the sampling wells was sampled after pumping. Parameters, including pH, dissolved oxygen (DO) concentration, electrical conductivity (EC), and temperature (*T*) were quantified with a portable water quality analyzer (Horiba D-24, Japan). The concentration of bicarbonate (HCO_3_^−^) was measured *in situ* using the titration kit (Merck, Germany). Water samples were filtered after sampling using a 0.22 μm cellulose acetate filter and then stored in precleaned polypropylene bottles. Major anions (Cl^−^, NO_3_^−^, and SO_4_^2−^) of filtered water samples were determined *via* ion chromatography (ICS 900, Dionex, United States). One aliquot of filtered water samples was acidified to pH < 2 with ultra-purified HNO_3_ for analyses of major cations (K^+^, Na^+^, Ca^2+^, and Mg^2+^) measurements using inductively coupled plasma-atomic emission spectrometry (ICP-AES, IRIS-HR, United States). The δ^18^O of ground water values were analyzed using a high-precision laser isotope analyzer (Picarro L2130-i Analyzer) with the measurement accuracy of 0.02%, and measurements were reported relative to the V-SMOW standard. δ^13^C_DIC_ values were analyzed using a MAT-253 mass spectrometer coupled with a Gas Bench II automated device with analytical precision of ±0.15%. The results are expressed as δ^13^C_DIC_ (%) with respect to the Vienna Pee Dee Belemnite (V-PDB) standard. Aqueous geochemical analyses and computational modeling provided saturation conditions for aquifer minerals, specifically calcite, dolomite and gypsum. The saturation index (SI) of a mineral is defined as log (IAP/Ksp), where IAP is the ion activity product and Ksp is a mineral thermodynamic equilibrium constant. A positive SI value indicates that precipitation with respect to a mineral is thermodynamically possible, whereas negative SI values indicate dissolution. A SI of 0 ± 0.5 indicates a mineral is at equilibrium within the solution (Gray and Engel, 2013). Deuterium excess (d-excess) is a second-order isotope parameter that is a function of the isotopic composition of oxygen and hydrogen in water, as defined by the Dansgaard’s equation: d-excess = δ^2^H − 8 × δ^18^O (Dansgaard, 1964).

### 2.2. Groundwater temperature data collection

The temperature–depth (TD) profiles of the six wells were determined using a COMPACT-TD logger (JFE Advantech, Japan), which simultaneously records water temperature and depth automatically; this equipment has a temperature resolution of 0.001°C and depth resolution of 0.008 m for every monitoring depth. Measurements were repeated to ensure that the equipment reached equilibrium with the surrounding environment (Li et al., 2019b). ‘Temperature-depth’ profile in the subsurface can be divided into the surficial zone and the geothermal zone (Parsons, 1970), and within the surficial zone temperature is influenced by seasonal heating and cooling of the land surface. Temperature profiles in the surficial zone potentially provide information about seasonal recharge/discharge events from precipitation and interchange with surface water (Anderson, 2005).

### 2.3. Microbiological analysis

For the microbiological analysis, 2 L water samples were filtered through polycarbonate filters (pore size = 0.22 μm). Total DNA was extracted from the water samples using the PowerSoil DNA Isolation Kit (MO BIO Laboratories, Carlsbad, CA, USA), according to the manufacturer’s instructions. The quality of the extracted DNA was checked using agarose gel electrophoresis and the DNA was stored at −20°C. DNA concentrations were determined using a Qubit® 2.0 fluorometer. Microcosms were constructed, deployed, and analyzed using a modified version of the method of Zhu et al. (2020). Briefly, the V4 region of bacterial 16S rRNA was amplified using primer pair 515F and 806R. PCR amplicons were sequenced on an Illumina MiSeq platform at Beijing Biomarker Technologies, and sequences were analyzed using QIIME and UPARSE software with the default settings to obtain effective tags and operational taxonomic units (OTUs). The UPARSE pipeline was then used for taxonomic assignment at the 97% similarity level *via* Ribosomal Database Project Naïve Bayesian Classifier v.2.2, trained on the SILVA database (ver. 123), using a 0.8 confidence level as the cutoff. The Mothur package was used to calculate the abundance-based coverage estimator (ACE) and Shannon’s diversity index.

### 2.4. n-Alkanes

To determine n-alkanes, the pH of the samples was adjusted to 2 and they were stored at 5°C until analysis. Methanol (10%) was added to the water samples before solid-phase extraction (SPE). n-Alkanes were constructed, deployed, and analyzed using a modification of the approach of Saim et al. (2009). n-Alkanes were extracted using C18 SPE cartridges conditioned with 10 ml of methanol followed by 6 ml of ultrapure water at a rate of 1–2 ml/min. The cartridges were then dried under vacuum. A 4 L water sample was loaded into the SPE column at a rate of 6 ml/min. The cartridges were then dried under vacuum for 30 min. The n-alkanes were eluted using 2 × 3 ml of dichloromethane. The extract was blown down to 1 ml under a gentle flow of nitrogen, and then analyzed by gas chromatography–mass spectrometry (GC–MS, Agilent 7890A GC, 5975C MSD) in selected ion monitoring (SIM) modes with internal standards n-Alkanes, and Deuterated tetracosane was used as internal standard for quantifying. The analysis was carried out in Chongqing Key Laboratory of Karst Environment, School of Geographical Sciences, Southwest University.

## 3. Results

### 3.1. Aquifer geochemistry

Borehole B1 had Ca–SO_4_-type water with total dissolved substances (TDS) > 1,000 mg/L; borehole B2 to B6 had Ca–HCO_3−_type water with TDS < 1,000 mg/L. The groundwater temperature varied from 23.4 to 27.1°C, i.e., did not vary markedly among boreholes; the pH ranged from 6.8 at B6 to 8.1 at B4. The d-excess also showed variation among wells, similar to the pH.

### 3.2. Taxonomic diversity

River water samples analyzed in this study was used to compare difference with groundwater samples. The coverage index of the sequenced samples ranged from 0.99 to 1.00; all values were >98%, indicating high reliability of the sequencing depth. The Shannon index was in the order B6 > B5 > B4 > B2 > R1 > B3 > B1, indicating a sequential decrease in microorganism diversity (Table 1). The OTUs obtained from the sequencing were analyzed taxonomically and represented 64 phyla, 200 classes, 370 orders, 540 families, 771 genera, and 814 species.

**Table 1:** Diversity and richness estimators for pyrosequence libraries.

Sample	Unique sequences	97% OTUs	Shannon	Coverage
R1	73,492	340	2.73	1.00
B1	69,975	1989	3.58	1.00
B2	76,178	1,084	3.98	1.00
B3	73,098	1769	3.94	0.99
B4	65,225	2,519	4.17	1.00
B5	74,911	1,081	4.99	1.00
B6	72,031	1,251	5.38	0.99

**Figure 2:**
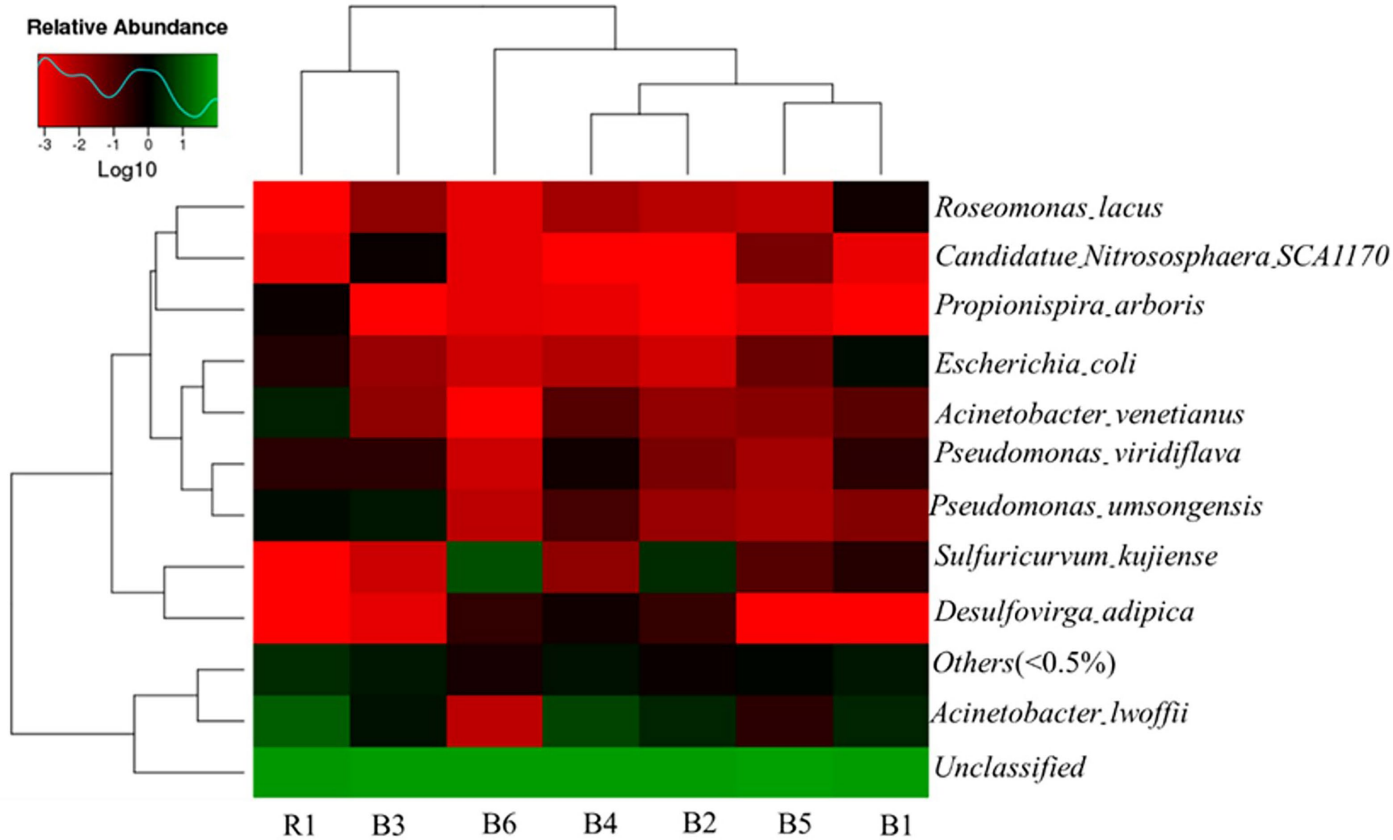
Heatmap of species relative abundance among different samples.

The distance measure used in CA (cluster analysis) was Pearson correlation, and the results are presented in a dendogram (Figure 2). As shown in Figure 2, the groundwater in B3 and river water in R1 were grouped together, groundwater in B4 and B2 belonged to same cluster, and B5 and B1 were grouped together. In addition, the species-level composition and abundance in groundwater *Acinetobacter lwoffii* was present in all water samples, except for B6. Its abundance in the environment was 0.27% ~ 7.21% in well water samples and 15.8% in river water samples, indicating that *A. lwoffii* was the dominant bacterium species in the studied samples. *S. kujiense* was dominant in B2 and B6, with respective abundances of 3.38 and 9.28%; similar lower abundances of *Desulfovirga adipica* were also presented in B2 and B6. However, *S. kujiense* and *Desulfovirga adipica* were not found in the river water sample (R1). *Acinetobacter venetianus*, *Pseudomonas umsongensis*, *Pseudomonas viridiflava*, and *Roseomonas lacus* were also presented.

### 3.3. n-Alkanes

**Figure 3:**
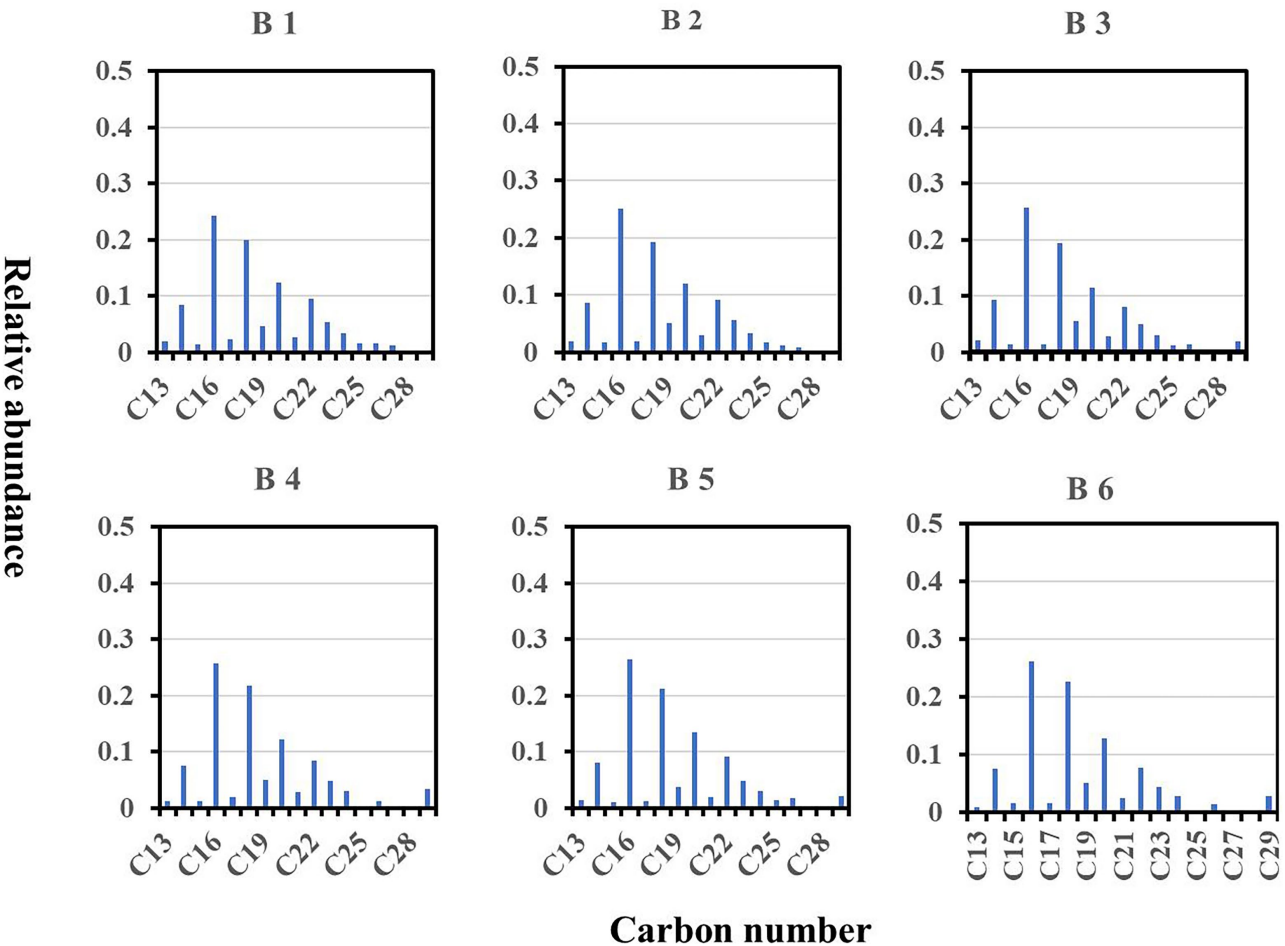
Concentration distributions of n-alkanes among wells.

The total dissolved n-alkane concentrations varied from 11,586 to 18,400 ng/l among the wells. Groundwater from all wells showed a unimodal distribution, with low-molecular-weight (LMW) n-alkanes having even numbers of carbons (n-C14 to n-C18) predominating; n-C16 was the dominant n-alkane (Figure 3). This indicates that the dissolved organic matter in the wells was of bacterial origin (Fang et al., 2014) and the dominant source of dissolved organic carbon in the aquifer is likely microbial primary production.

## 4. Discussion

### 4.1. Hydrodynamic characteristics among different karst aquifer system

**Figure 4:**
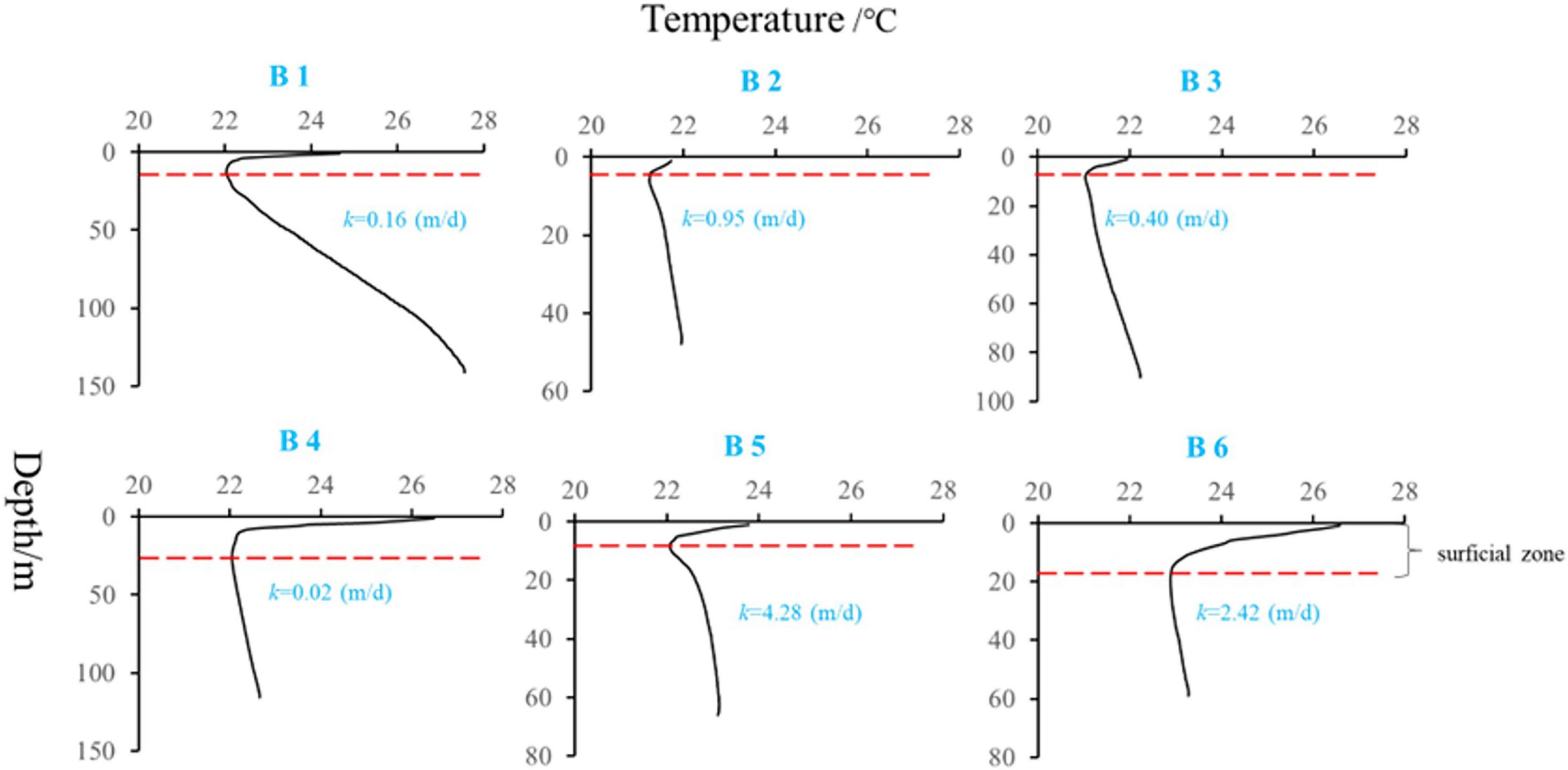
‘Temperature-depth’ profiles of the selected boreholes (k, hydraulic conductivity; red dashed line, surficial zone boundary).

Heat carried by groundwater serves as a tracer to identify flow through fractures, and flow patterns in groundwater basins (Anderson, 2005), and the groundwater flow in the preponderance flow path interferes with the normal temperature distribution of the formation, and the information of groundwater seepage in the formation can be inferred from the anomaly of the temperature curve (Chi et al., 2020). In this study, six boreholes ‘temperature-depth’ profile were used to characterize the hydraulic properties of aquifers, the temperature gradient in B1, B2, B3, B4, B5, and B6 were 4.3, 1.7, 1.5, 0.6, 1.8, and1.0°C/100 m, respectively (Figure 4). Among the selected boreholes, B1 belongs to non-karst aquifer and gypsum interlayer grows on it, which can also be reflected from the high SO_4_^2−^ concentration in groundwater (Table 2). It also be found that lower temperature gradient was also corresponded to higher *k* values in karst aquifers (especially in B2, B3, B5 and B6).

**Table 2:** Chemical and physical properties among different wells.

Item	River	Well					
	R1	B1	B2	B3	B4	B5	B6
TDS(mg/L)	437	2,652	499	377	164	304	409
DO(mg/L)	3.2	2.1	1.9	2.2	2.1	1.8	1.5
T(°C)	25	25.8	27.1	23.4	23.6	25.8	25.6
Ph	6.9	7.3	6.9	7.5	8.1	7.7	6.8
DOC(mg/L)	0.9	0.7	0.6	0.5	0.5	1	1.6
d-excess (%)	13.3	11.3	7.8	13.1	12.8	11.7	10.1
δ^13^C_DIC_ (%)	-	−8.19	−7.88	−8.09	−4.65	−7.88	−8.66
^3^H (TU)	-	< 2	3.44	8.27	2.78	3.16	6.61
HCO_3_^−^(meq/L)	4	3	7.2	4	2.4	3.2	4.3
Cl^−^(meq/L)	0.9	0.2	0.1	0.1	0	0.1	0.2
NO_3_^−^(meq/L)	1	BDL	0	0	0.1	0.1	BDL
SO_4_^2−^(meq/L)	0.4	42	0.3	0.5	0.2	0.5	0.6
K^+^(meq/L)	0.4	0.1	0	0	0	0	0.1
Na^2+^(meq/L)	0.8	1.7	0.1	0	0	0	0.3
Cl^−^(meq/L)	0.9	0.2	0.1	0.1	0	0.1	0.2
Ca^2+^(meq/L)	4.7	17	1.8	4.3	0.3	3.9	4.4
Mg^2+^(meq/L)	0.2	5.7	0.2	1.4	0.1	0.4	0.7

DO, dissolved oxygen; BDL, below detection limit; DOC, dissolved organic carbon; d-excess, deuterium excess; −, missing data.

In the karst aquifer system, there are groundwater migration channels such as pores, fissures, and cavities with large diameters. In areas where karst is not formed, rock mass is dense, and small pores are dominant, groundwater flow rate is very slow, even water-tight. It can be concluded that from borehole B2 to B6, karst aquifers are featured by vertical flow and have the characteristics of groundwater seepage. Especially, in the light of borehole profile description between wells (Supplementary Figure S1), the karst aquifer in B5 showed high permeability, karstification was well developed, and fissures and caves were found. By contrast, aquifer in B1 was not karst aquifer, and the vertical flow rate is slow. In addition, groundwater age estimation using tritium (^3^H) only provides semi-quantitative values (Brkic et al., 2016): <0.8 TU indicates sub-modern water (recharged prior to 1950s), 0.8 to ~4 TU indicates a mix of sub-modern and modern water, 5 to 15 TU indicates modern water (<5 to 10 years),15–30 TU indicates some bomb tritium and > 30 TU indicates recharged occurred in the 1960s to 1970s. As shown in Table 2, B3 and B6 had ^3^H values higher than 5 TU, indicating modern water (< 5 to 10 years) in above aquifers; whereas ^3^H values in B2, B4 and B5 all higher than 0.8 TU and lower than 4 TU, showing a mix of sub-modern and modern water in above aquifers. And B1 showing ^3^H values lower than 2, which may indicate sub-modern water in it. Hence, combined with the ‘temperature-depth’ profile characteristics and ^3^H values above in selected boreholes, it can conclude that karst aquifer in borehole B1 belongs to regional flow, karst aquifers in borehole B3 and B6 perhaps belong to local flow, and karst aquifers in borehole B2, B4 and B5 maybe belong to intermediate flow. In addition, similar distribution of microbe species between river water and groundwater in B3 also support that groundwater in B3 was more potentially influenced by seepage, and which should belong to local flow.

### 4.2. Potential factors influencing microbe activities in selected karst aquifers

Principal Component Analysis (PCA) is based on the diagonalization of the correlation matrix, which can not only point out associations between variables that can show the global coherence of the data set, but also it will evidence the participation of the individual chemical parameters in several influence factors (Helena et al., 2000), and help to explore the dominating factors in the geochemistry (Hu et al., 2013). In this study, PCA was performed to identify potential factors influencing microbe activities in selected karst aquifers, which is a common phenomenon in hydrochemistry.

In the PCA, the first three eigenvalues were greater than one and explained more than 88% of the variance (Table 3). The first principal component (PC1) accounted for more than 47.9% of the variance in the data and had high positive loadings for *A*. *lwoffii*, *A*. *venetianus*, *Escherichia coli*, *Propionispira arboris*, EC, DO, Cl^−^, NO_3_^−^, K^+^, and Na^+^. In groundwater, *E*. *coli* and NO_3_^−^ are usually from the input of effluent (Dougherty et al., 2009), while K^+^ and Na^+^ ions can result from agricultural fertilizer use in rural and suburban areas, as well as from livestock manure and sewer leakage (Liang et al., 2018). This indicates that *A*. *lwoffii*, *A*. *venetianus*, *E*. *coli*, and *P*. *arboris* were not from the internal environment of the karst aquifer.

**Table 3:** The main component load of bacterial species and their water physics and chemical parameters.

Items	PC 1	PC 2	PC 3
*Acinetobacter_lwoffii*	**0.858**	0.21	−0.338
*Acinetobacter_venetianus*	**0.995**	0.047	−0.064
*Candidatus_Nitrososphaera_SCA1170*	−0.243	0.39	**0.881**
*Desulfovirga_adipica*	−0.423	0.256	**−0.717**
*Escherichia_coli*	**0.995**	0.041	−0.057
*Propionispira_arboris*	**0.997**	0.032	−0.046
*Pseudomonas_umsongensis*	0.452	0.432	**0.752**
*Pseudomonas_viridiflava*	0.032	**0.819**	−0.329
*Roseomonas_lacus*	−0.552	0.267	0.547
*Sulfuricurvum_kujiense*	−0.259	**−0.868**	−0.052
EC	**0.645**	−0.491	0.492
DO	**0.943**	0.258	0.024
Temperature	0.039	**−0.971**	−0.179
pH	−0.555	**0.714**	−0.316
DOC	0.547	−0.302	−0.209
d-excess	0.373	**0.895**	0.12
HCO_3_^−^	−0.03	**−0.87**	0.253
Cl^−^	**0.999**	0.007	−0.031
NO_3_^−^	**0.994**	0.047	−0.09
SO_4_^2−^	0.118	0.009	**0.85**
K^+^	**0.995**	−0.047	−0.052
Na^+^	**0.989**	−0.092	−0.034
Eigenvalue	10.55	5.37	3.62
Total variance (%)	47.94	24.41	16.47
Cumulative variability (%)	47.94	72.35	88.82

The second principal component (PC2) accounted for more than 24.4% of the variance and had high loadings for *P*. *viridiflava*, pH, and d-excess (all positive) and *S*. *kujiense*, temperature, and HCO_3_^−^ (all negative). *S*. *kujiense* is a parthenogenetic, anaerobic, chemoautotrophic sulfur-oxidizing bacterium, and temperature and pH control sulfur-oxidizing bacterium. This indicates that the HCO_3_^−^ concentration increased with the density of *S*. *kujiense*.

The third principal component (PC3) accounted for more than 16.47% of the variance and had high loadings for *P*. *umsongensis*, *Candidatus Nitrososphaera SCA1170*, and SO_4_^2−^ (all positive) and *D*. *adipica* (negative), and moderate loading for *R*. *lacus*. This indicates that the SO_4_^2−^ concentration increased with the relative abundance of *Candidatus_Nitrososphaera_SCA1170* and *P*. *umsongensis*, but decreased with the relative abundance of *D*. *adipica*.

**Figure 5:**
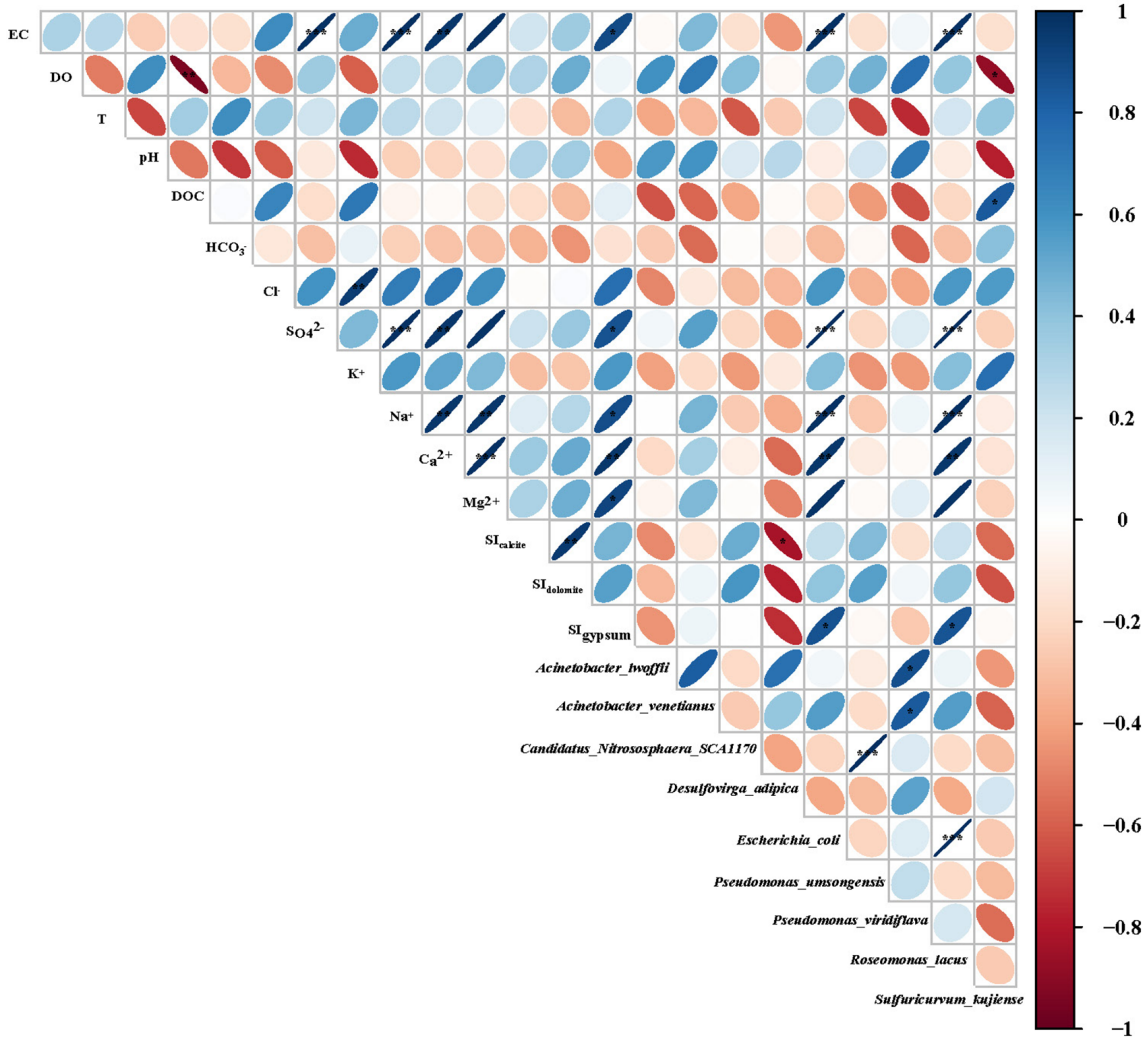
Correlation heatmap for groundwater environmental parameters and microbe species (In brief, orange and red colors indicate negative correlations, blue colors indicate positive correlations and the strength of color indicates the magnitude of correlation coefficient, as shown on the correlation spectrum, insert right; ^*^p < 0.05, ^**^p < 0.01, ^***^p < 0.001).

In this study, the length of the gradient in the first axis calculated by detrended correspondence analysis (DCA) was <3, which was 2.8 in our study, the redundancy analysis (RDA) model should be selected to evaluate the potential relationship between the distribution of microbe species and environmental parameters (Chen et al., 2015), but the *p* values of environmental factors in groundwater were all higher than 0.05, which can not be used to evaluate the potential relationship between the distribution of microbe species and environmental parameters in our study. Hence, the relationship between microbe species abundance and groundwater environmental factors in six selected boreholes was evaluated by multivariate analysis (Figure 5).

As shown in Figure 5, similar relationships were found between selected microbe species (*E. coli* and *R. lacus*) and groundwater environmental factors (K^+^, Na^+^, Ca^2+^, Mg^2+^, and SI_dolomite_), indicating *E. coli* and *R. lacus* may produce influence on groundwater chemistry types. And also, *S. kujiense* showed higher positive relationship with HCO_3_^−^ (*p* < 0.05), indicating the abundance of *S. kujiense* in karst groundwater was an important factor that influencing HCO_3_^−^ concentrations.

**Figure 6:**
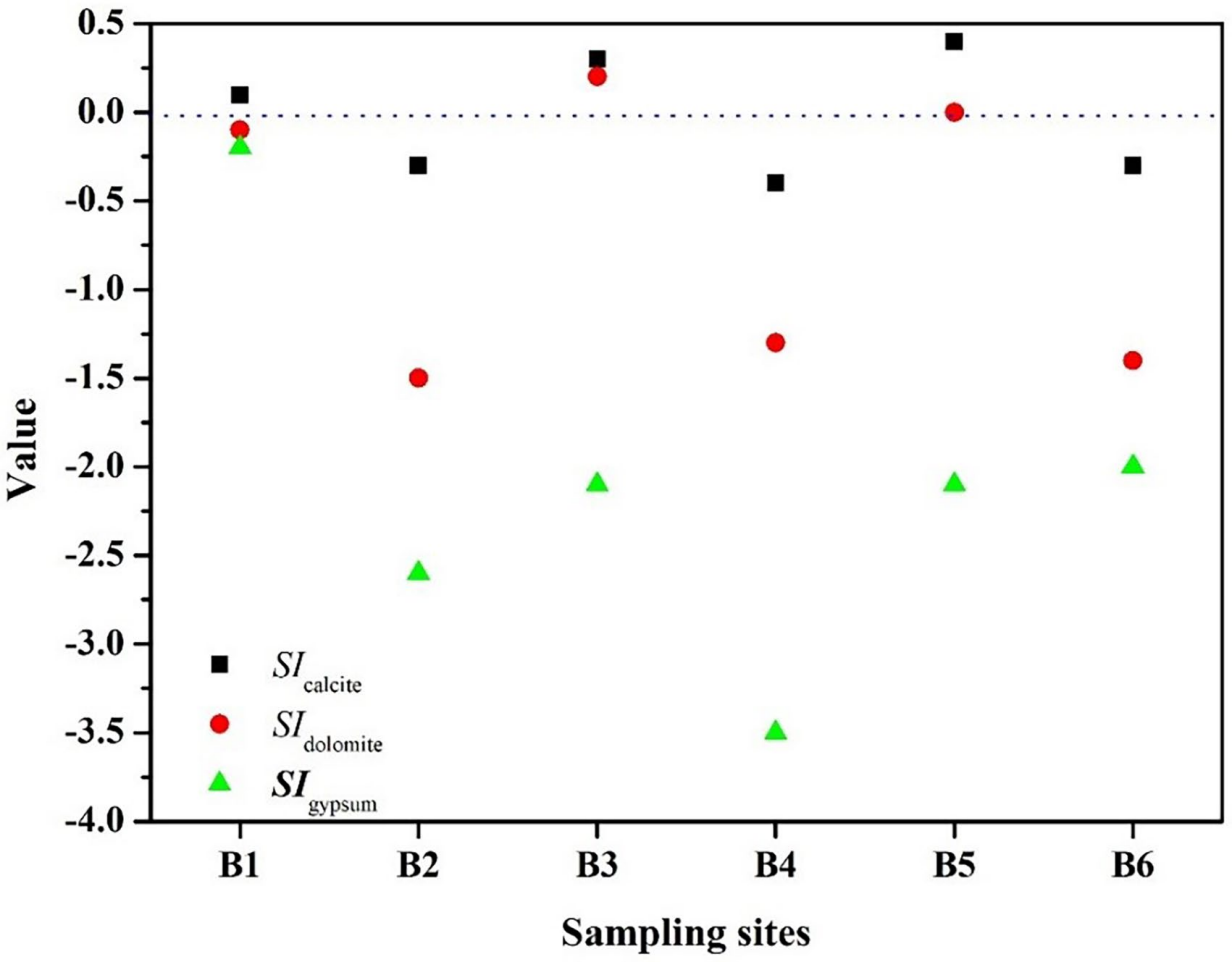
Saturation indexes of the major minerals at the different boreholes. The relative abundance of *S. kujiense* among the different wells roughly matched the HCO_3_^−^ concentrations (Figure 5), especially in B2 and B6 (Figure 7).

**Figure 7:**
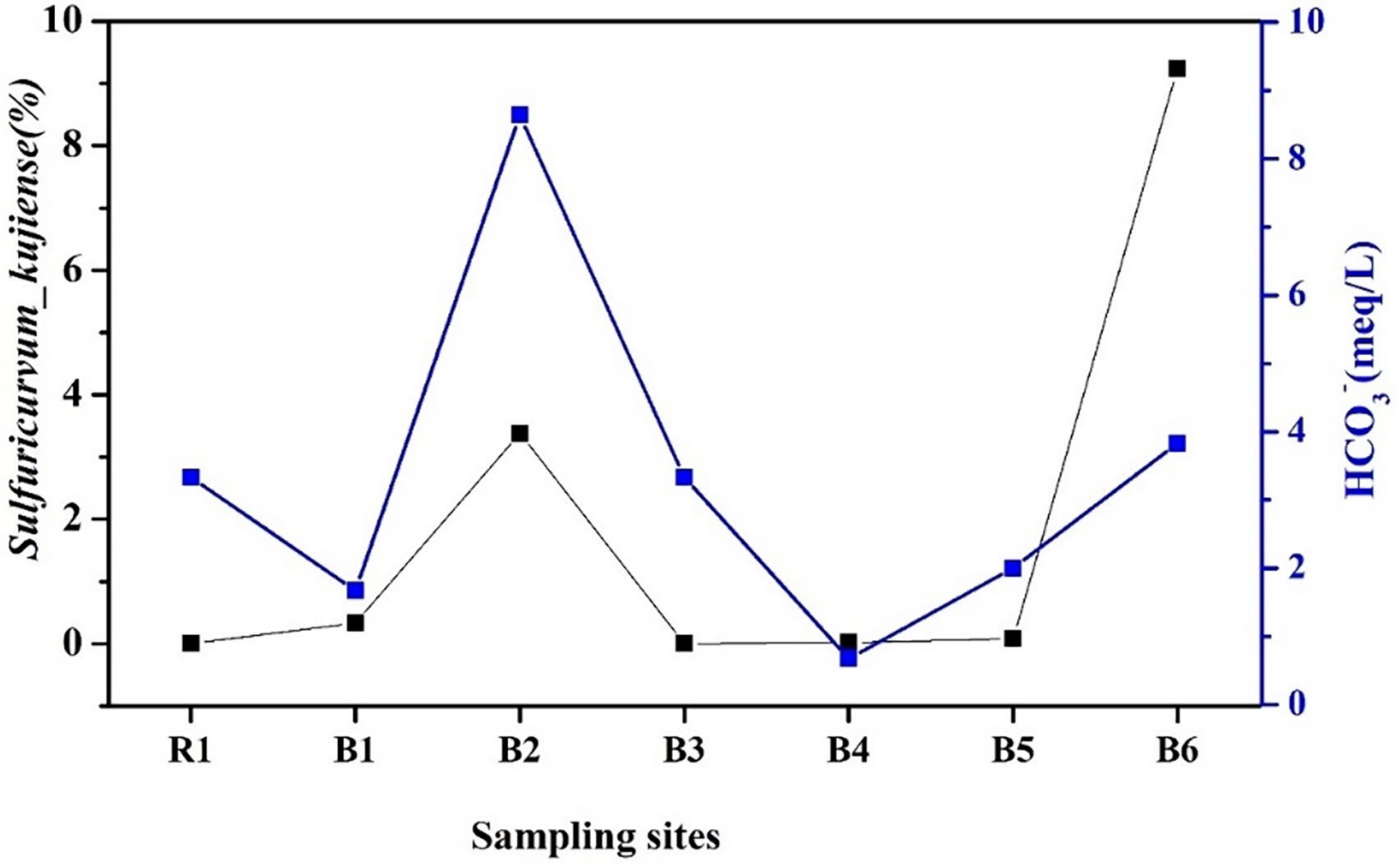
Variation in bicarbonate concentrations and the relative abundance of bacterial species at different boreholes.

Because the HCO_3_^−^ concentration increased with the density of *S*. *kujiense*. It is necessary to assess the solubility of carbonate minerals based on bulk aquifer fluid geochemistry before considering the potential for microbially mediated carbonate dissolution. By calculating the saturation index (SI), the equilibrium state of water with respect to a particular mineral phase can be determined, and which can also be used to distinguish the hydrogeochemical evolution as well as by identifying the geochemical reactions that control the water chemistry (Li et al., 2019a; Subba Rao et al., 2022). The saturation indexes of calcite, dolomite, and gypsum were calculated to interpret the hydrogeochemical processes in the six wells (Figure 5). The saturation indexes of calcite, dolomite and gypsum in B1 were all around 0, indicating hydrogeochemical processes at B1 was saturated state; B2, B4, and B6 were undersaturated with respect to calcite, dolomite, and gypsum and had higher relative abundances of *S*. *kujiense* (B2, 3.38%; B4, 0.02%; B6, 9.23%), while the saturation indexes of calcite, dolomite, and gypsum were all less than 0 in B2, B4 and B6, indicating an unsaturated state in all groundwaters. Besides, the saturation indexes of gypsum in B3 and B5 were all less than 0, indicating unsaturated state, but the saturation indexes of calcite and dolomite were all higher than or equal to 0, indicating ‘rapid dissolution’ to ‘dissolution equilibrium’ state. In multiple groundwater flow systems, related study found that carbonate dissolution dominated the local flow, carbonate dissolution and gypsum dissolution coexisted in the intermediate flow, and gypsum dissolution dominated the regional flow (Wang et al., 2022). Combined with the groundwater flow characteristics based on ‘temperature-depth’ profile characteristics and age dating of the boreholes (Figure 4), it can be concluded that the karst aquifers in B3 and B6 (which belong to local flow) have higher saturation indexes (with the saturation indexes of calcite and dolomite all higher than 0); the karst aquifers in B2, B4 and B5 (which belong to intermediate flow) have lower saturation indexes (with the saturation indexes of calcite and dolomite all less than 0); aquifer in borehole B1 (which belong to regional flow) with the saturation indexes of calcite, dolomite and gypsum all around 0 (Figures 6, 7).

In groundwater, d-excess reflects the extent of water/rock oxygen isotope exchange on a regional scale and indicates its variability, the stronger the water/rock interaction the lower the d-excess (Liu et al., 2016). It was listed in Table 2 that, higher d-excess values were presented in B3, B4 and B5, while lower d-excess values were found in B2 and B6, which indicated that the stronger water/rock interaction were happened in B2 and B6. Study found sulfur-oxidizing bacteria consume reduced-sulfur compounds and produce sulfuric acid, which decreases the surface pH locally and promotes carbonate dissolution near a shallow groundwater table (Macalady et al., 2006). They can also influence aquifer-scale geochemical processes because they are metabolically active at low oxygen tensions under completely anaerobic conditions (Goldscheider et al., 2006). Microbially promoted carbonate dissolution is enhanced by a local decrease in pH at the cell–mineral interface (Sjöberg and Rickard, 1984; MacInnis and Brantley, 1992), and *S*. *kujiense* is a parthenogenetic, anaerobic, chemoautotrophic sulfur-oxidizing bacterium, the temperature and pH control sulfur-oxidizing bacterium, therefore, lower pH values in B2 and B6 perhaps enhance the carbonate dissolution medicated by *S*. *kujiense*, and lower d-excess values in B2 and B6 reflected the higher degree of carbonate dissolution medicated by *S*. *kujiense.*

n-Alkanes of bacterial origin are characterized by a predominance of even numbers of carbon atoms (14, 16 or 18; Derrien et al., 2017), which matches our results that the organic matter in the studied wells is predominately caused by autochthonous microbial activity and seldom no allochthonous organic matter input. Because *S. kujiense* is a chemoautotrophic sulfur-oxidizing bacterium, which is no need to require organic substrates to get their carbon for growth and development in karst aquifers. Which also supports that *S. kujiense* potentially mediated carbonate dissolution in the karst aquifers of B2 and B6, and it should be noted that higher dissolution and caves in karst aquifer were also found in B6 (Supplementary Figure S1).

In addition to the effects of microbial activity on karst aquifers, chemical reactions play an important role in internal processes. For instance, carbonic acid and anthropogenic acids (e.g., sulfuric and nitric acid) increase the dissolution of carbonate and HCO_3_^−^ concentration in groundwater (Huang et al., 2017). Carbon isotopes in groundwater (δ^13^C_DIC_) can be used to identify different acids affecting carbonate dissolution (Ali and Atekwana, 2011). In the study area, the rainwater was typically acidic, with a volume-weighted mean pH of 4.49 (Cao et al., 2009), which indicates that atmospheric CO_2_ makes a negligible contribution to dissolved inorganic carbon (DIC). The DIC in the study area likely has two primary sources: the weathering of carbonate minerals and the dissolution of CO_2_ in soil (Eq. 1).


(1) $$\begin{array}{l} Ca_{\left(1-x\right)}Mg_{x}CO_{3}+H_{2}CO_{3}\\ =\left(1-x\right)Ca^{2+}+xMg^{2+}+2HCO_{3}^{-} \end{array}$$

Open-system carbonate weathering mediated solely by carbonic acid requires that the isotopic composition of DIC is continuously in equilibrium with the gaseous phase of a given pCO_2_, and that continuous isotopic exchange occurs between CO_2_ and the aqueous solution. Thus, δ^13^C_DIC_ is controlled mainly by the hydrolysis of CO_2_ in soil (Jiang et al., 2013; Huang et al., 2017). Therefore, the δ^13^C_DIC_ in karst groundwater under open-system conditions should be around −14% (i.e., −23% plus +9%; Jiang et al., 2013). In a closed-system, the amount of soil-derived CO_2_ decreases gradually over time during carbonic acid-driven carbonate dissolution; as the carbon in our groundwater samples was produced from carbonate and soil-derived CO_2_ in approximately equal amounts, the δ^13^C_DIC_ should approach a value of −11.5% [i.e., 0.5 × (−23% + 0)] (Jiang et al., 2013). When carbonate dissolution is facilitated by other acids (e.g., sulfuric, nitric, or organic acid), all DIC is derived from non-carbonic acid carbonate dissolution (Eq. 2), so it has a δ^13^C value (0%) identical to that of the constituent carbonate minerals (Li et al., 2008; Jiang et al., 2013).


(2) $$\begin{array}{l} 3Ca_{\left(1-x\right)}Mg_{x}CO_{3}+H_{2}SO_{4}+HNO_{3}⇌3\left(1-x\right)Ca^{2+}\\ +3xMg^{2+}+SO_{4}^{2-}+NO_{3}^{-}+3HCO_{3}^{-} \end{array}$$

For the groundwater analyzed, the δ^13^C_DIC_ values were in the range − 8.66% to −4.65% with a mean value of −7.56%, which is significantly more positive than −11.5% (Table 2). This suggests that carbonate dissolution in the study area was likely facilitated by additional acids. Because groundwater recharge areas have vast areas of fertile agricultural land, higher levels of domestic sewage and nitrogenous fertilizers percolate through the topsoil and enter the karst aquifers, which can increase carbonate dissolution (Yamanaka, 2012). The larger range of δ^13^C_DIC_ values is strongly related to enhanced carbonate dissolution by nitric and sulfuric acids.

## 5. Conclusion

The main conclusion of this study is that carbonate dissolution in the deep karst aquifer was potentially influenced by microbial activity (e.g., *S*. *kujiense*) and the input of anthropogenic acids, as evidenced by significantly more positive δ^13^C_DIC_ values. This ultimately changes the hydraulic properties of karst aquifers. However, more studies are needed to quantify the effect of microbial activity on carbonate dissolution in the karst aquifer.

## Data availability statement

The data presented in the study are deposited in the NCBI repository, accession number PRJNA917210.

## Author contributions

ZL wrote the manuscript. SL, ZW, RL, and ZY performed the data collection and the bioinformatic analysis. JC and LG conceived the idea and supervised the work. YS performed the lipids biomarker analysis. All authors contributed to the article and approved the submitted version.

## Funding

This work was financially supported by Natural Science Foundation of Guangdong Province of China (2021A1515110505), Open Funding Project of the Key Laboratory of Groundwater Sciences and Engineering, Ministry of Natural Resources (SK202102), National Natural Science Foundation of China (41961144027 and 41771027), China Postdoctoral Science Foundation (2021M703657), Scientific and Technological Innovation Project of the Water Sciences Department of Guangdong Province (2020–09), and Asia-Pacific Network for Global Change Research (APN; CRRP2019-09MY-Onodera).

## Conflict of interest

The authors declare that the research was conducted in the absence of any commercial or financial relationships that could be construed as a potential conflict of interest.

## Publisher’s note

All claims expressed in this article are solely those of the authors and do not necessarily represent those of their affiliated organizations, or those of the publisher, the editors and the reviewers. Any product that may be evaluated in this article, or claim that may be made by its manufacturer, is not guaranteed or endorsed by the publisher.
