# Supplementary material for: Microbial community structure characteristics among different karst aquifer systems, and its potential role in modifying hydraulic properties of karst aquifers
Source: Front Microbiol. 2023 Jan 17;13:1054295. doi: 10.3389/fmicb.2022.1054295 (PMC9887151; doi:10.3389/fmicb.2022.1054295)
Supplement: Supplementary file 1 [file Image_1.pdf]

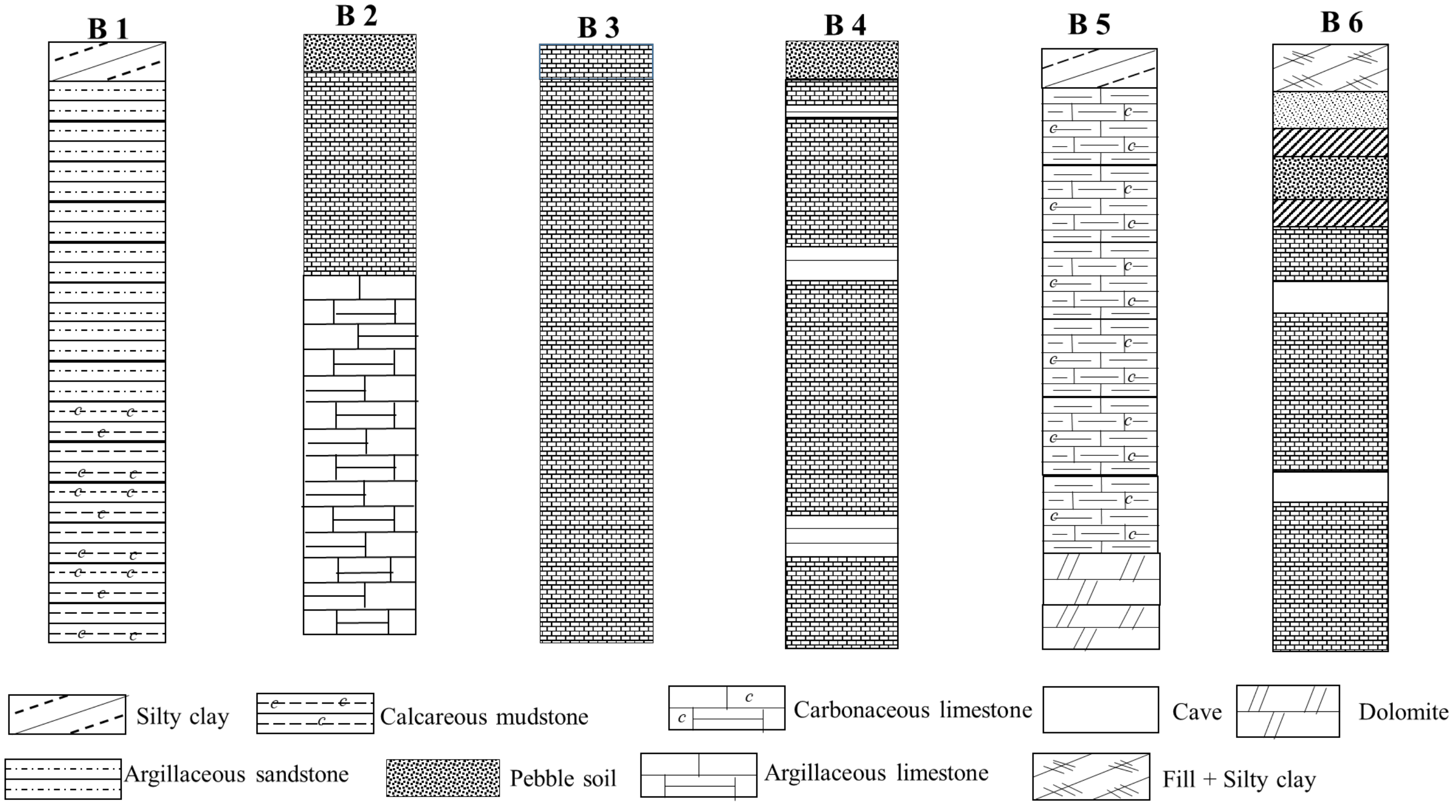

Figure S1. Lithological characteristics between selected boreholes

(**B1**, low permeability and low water richness; no fissures developed; **B2**, joints and fissures relatively well-developed, dissolution occurs in the upper aquifer; **B3**, joints and fissures not developed, dissolution only occurs in the top of the aquifer; **B4**, joints and fissures relatively developed, dissolution is obvious, and caves were found; **B5,** joints and fissures are not developed, dissolution is no obvious; **B6,** caves are well-developed in the aquifer)
