# Supplementary material for: Microbial community structure characteristics among different karst aquifer systems, and its potential role in modifying hydraulic properties of karst aquifers
Source: Front Microbiol. 2023 Jan 17;13:1054295. doi: 10.3389/fmicb.2022.1054295 (PMC9887151; doi:10.3389/fmicb.2022.1054295)
Supplement: Supplementary file 2 [file Table_4.DOCX]

Table **S1** Information about selected boreholes

|  | Stratum | Lithology | Well depth (m) | Aquifer thickness (m) |
| --- | --- | --- | --- | --- |
| B1 | Cretaceous (K*n*) | Calcareous mudstone | 100.6 | 17 |
| B2 | Devonian (D*b+lj*) | Argillaceous limestone | 80.6 | 9.5 |
| B3 | Devonian (D*r*) | Limestone | 100.05 | 8.7 |
| B4 | Carboniferous (Cs) | Limestone | 108.7 | 28.77 |
| B5 | Carboniferous (Cs) | Dolomite | 100.2 | 34.3 |
| B6 | Devonian (D*_3_t*) | Limestone | 65.7 | 8.5 |
